# The prevalence and anatomical characteristics of the accessory head of the flexor pollicis longus muscle: a meta-analysis

**DOI:** 10.7717/peerj.1255

**Published:** 2015-10-01

**Authors:** Joyeeta Roy, Brandon M. Henry, Przemysław A. Pękala, Jens Vikse, Piravin Kumar Ramakrishnan, Jerzy A. Walocha, Krzysztof A. Tomaszewski

**Affiliations:** Department of Anatomy, Jagiellonian University Medical College, Krakow, Poland; International Evidence-Based Anatomy Working Group, Krakow, Poland

**Keywords:** Gantzer’s muscle, Accessory head, Meta-analysis, Anatomy, Flexor pollicis longus

## Abstract

**Background and Objectives.** The accessory head of the flexor pollicis longus muscle (AHFPL), also known as the Gantzer’s muscle, was first described in 1813. The prevalence rates of an AHFPL significantly vary between studies, and no consensus has been reached on the numerous variations reported in its origin, innervation, and relationships to the Anterior Interosseous Nerve (AIN) and the Median Nerve (MN). The aim of our study was to determine the true prevalence of AHFPL and to study its associated anatomical characteristics.

**Methods.** A search of the major electronic databases PubMed, EMBASE, Scopus, ScienceDirect, and Web of Science was performed to identify all articles reporting data on the prevalence of AHPFL in the population. No date or language restriction was set. Additionally, an extensive search of the references of all relevant articles was performed. Data on the prevalence of the AHFPL in upper limbs and its anatomical characteristics and relationships including origin, insertion, innervation, and position was extracted and pooled into a meta-analysis using MetaXL version 2.0.

**Results.** A total of 24 cadaveric studies (*n* = 2,358 upper limb) were included in the meta-analysis. The pooled prevalence of an AHFPL was 44.2% (95% CI [0.347–0.540]). An AHFPL was found more commonly in men than in women (41.1% vs. 24.1%), and was slightly more prevalent on the right side than on the left side (52.8% vs. 45.2%). The most common origin of the AHFPL was from the medial epicondyle of the humerus with a pooled prevalence of 43.6% (95% CI [0.166–0.521]). In most cases, the AHFPL inserted into the flexor pollicis longus muscle (94.6%, 95% CI [0.731–1.0]) and was innervated by the AIN (97.3%, 95% CI [0.924–0.993]).

**Conclusion.** The AHFPL should be considered as more a part of normal anatomy than an anatomical variant. The variability in its anatomical characteristics, and its potential to cause compression of the AIN and MN, must be taken into account by physicians to avoid iatrogenic injury during decompression procedures and to aid in the diagnosis and treatment of Anterior Interosseous Nerve Syndrome.

## Introduction

The flexor pollicis longus (FPL) muscle originates from the anterior part of the radius and the anterior interosseous membrane, below the anterior oblique line and above the insertion of the pronator quadratus, to insert into the distal phalanx of the thumb (Dolderer et al., 2011; El Domiaty, Zoair & Sheta, 2008). Receiving innervation from the anterior interosseous nerve (AIN), a branch of the median nerve (MN), it is crucial for flexion of the interphalangeal joints of the thumb (Dolderer et al., 2011). The FPL has long been associated with a tendinous slip or an occasional muscular head that joins the FPL with the deep flexors of the finger (Caetano et al., 2015; Wood, 1868).

The upper limb muscles originate in the fourth week of development from the somatic mesoderm. The mesoderm invades the limb buds to form dorsal and ventral condensations, which give rise to the upper limb pronators and flexors, respectively. The flexors further develop from a flexor mass, dividing into superficial and deep layers (El Domiaty, Zoair & Sheta, 2008). The deep layer gives rise to the FPL, flexor digitorum profundus (FDP) and flexor digitorum superficialis (FDS). The incomplete cleavage of the flexor mass during development has been thought to give rise to the accessory muscle (Kara et al., 2012; Jones et al., 1997).

**Figure 1:**
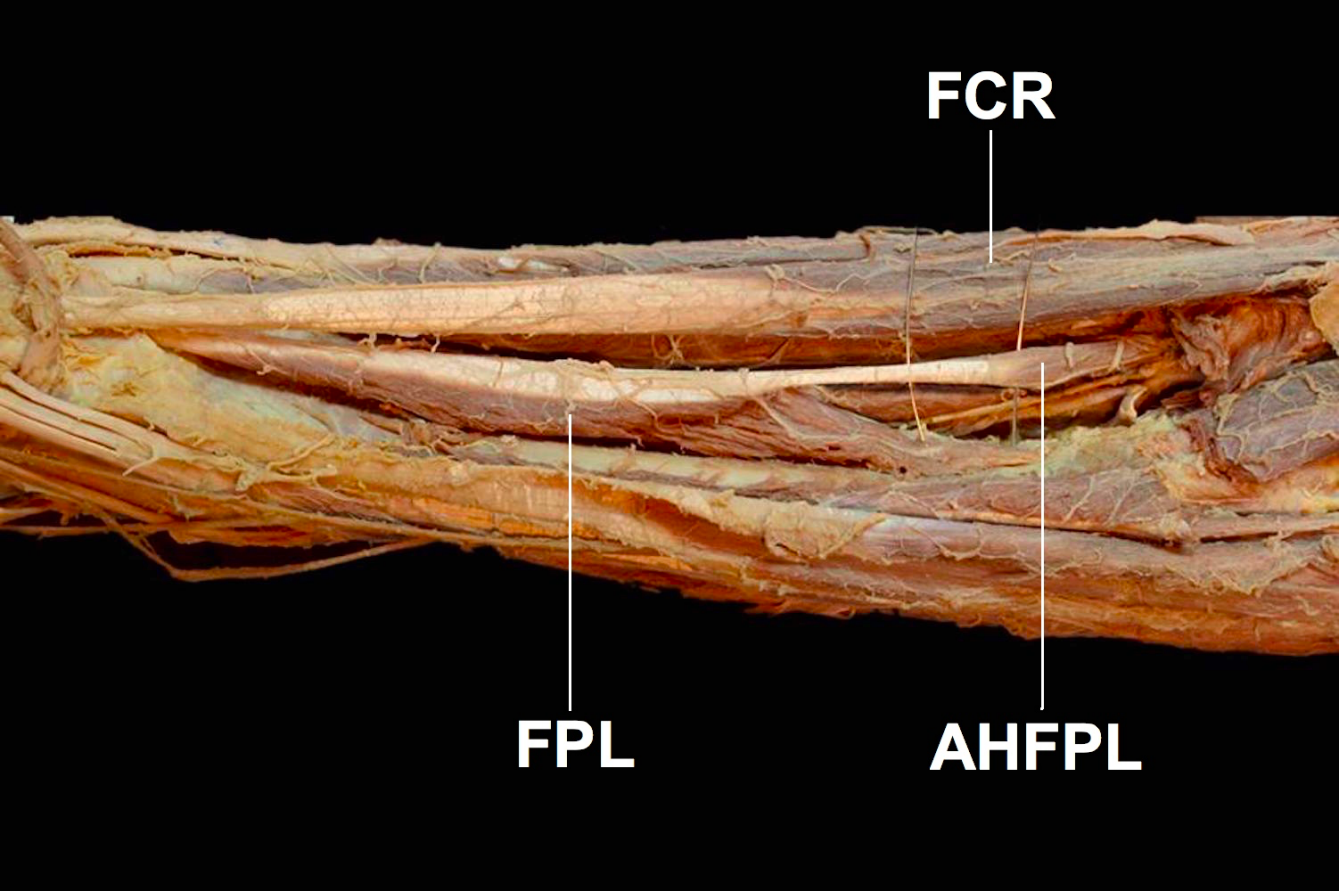
The accessory head of the flexor pollicis longus muscle (AHFPL) originating from the flexoe digitoum profundus. AHFPL, accessory head of flexor pollicis longus; FPL, flexor pollicis longus; FCR, flexor carpi radialis.

First described in 1813 and dubbed eponymously by Gantzer as Gantzer’s muscle, the status of the accessory head of the flexor pollicis longus muscle (AHFPL) as an anatomical variant or a normal anatomical structure has been widely debated (Fig. 1) (Caetano et al., 2015). The AHFPL is considered by many to be an anatomical variant whose prevalence rates vary significantly among populations examined by different studies. Apart from the differing prevalence rates, there is also discordance among authors about the origin of the muscle, ranging from the coronoid process of the ulna (Jones et al., 1997) to the medial epicondyle of the humerus (Dykes & Anson, 1944). While the insertion of the muscle is more widely accepted as being into the FPL, authors are still divided about whether it inserts into the upper, middle or lower thirds of the FPL tendon (Gunnal et al., 2013). Furthermore, there is also disagreement about the innervation of the AHFPL, with authors suggesting the muscle receives innervation from the AIN, the MN or dual innervation from both the AIN and MN (Jones et al., 1997). This disagreement extends further into the position of the AHFPL in relation to the AIN and MN, with studies reporting the muscle lying in between the MN anteriorly and the AIN posteriorly (Mangini, 1960; Hemmady, Subramanya & Mehta, 1993) or the AHFPL lying posterior to both nerves (Dellon & Mackinnon, 1987; Al-Qattan, 1996; Oh, Chung & Koh, 2000). Although controversial, these variable anatomical and topographical relationships the AHFPL has with the MN and AIN, confers the possibility of the muscle to be an important source of compression of these nerves with potential clinical significance, such as Anterior Interosseous Nerve Syndrome (AINS) or pronator teres syndrome (Gunnal et al., 2013).

Compression neuropathies in the proximal forearm present with vague symptoms and are notoriously difficult to diagnose, resulting in patients going undiagnosed for months or even years (Gunnal et al., 2013). Anterior Interosseous Nerve Syndrome usually presents as acute pain that eventually subsides over hours or days, followed by paresis or paralysis of the FPL, FDP and pronator quadratus (PQ) muscles (Gunnal et al., 2013). AINS is divided into a complete type, where the whole nerve passes posterior and underneath the AHFPL resulting in loss of function of the FDP, FPL and PQ muscles, and an incomplete type, where the medial branch which innervates the FDP passes underneath the AHFPL and is compressed causing an isolated paralysis of the FPL with a characteristic pinch movement disability (Kara et al., 2012; Gunnal et al., 2013). Furthermore, the AHFPL has also been implicated in MN compression leading to paralysis of the thenar muscles and loss of sensation in the hand (Caetano et al., 2015).

Taking into account the clinical relevance of the AHFPL, the aim of our study was to determine the true population prevalence rate of the AHFPL, and to study its associated anatomical characteristics.

## Materials and Methods

### Search strategy

To identify articles eligible for inclusion into the meta-analysis, an extensive search of the following electronic databases was performed up to May 2015: PubMed, EMBASE, Scopus, ScienceDirect, and Web of Science. The search terms included: Gantzer’s muscle, accessory head of flexor pollicis longus, and AHFPL. No date or language restrictions were set. To further identify all potentially eligible articles for the meta-analysis, an extensive search of the references of all relevant articles was also performed. Preferred Reporting Items for Systematic Reviews and Meta-analyses (PRISMA) guidelines were strictly followed during the search process and throughout the entire meta-analysis (Supplemental Information 1).

### Criteria for study selection

Assessment of eligibility for inclusion into the meta-analysis was performed by two reviewers (JR and BMH). Studies were considered eligible for inclusion in the meta-analysis if they reported extractable data on the prevalence of AHFPL in upper limbs. The exclusion criteria for the meta-analysis included (1) if the articles were case reports, letters to the editor, and conference abstracts, (2) the study reported incomplete data, or (3) reported prevalence data with respect to rate per cadavers. Studies that were published in languages not spoken fluently by any of the authors, were translated by medical professionals fluent in both English and the language of the manuscript. Any disagreements among the reviewers during the eligibility assessment were solved by a consensus among all the reviewers.

### Data extraction

Data was independently extracted by two reviewers (JR and PP) from the included studies. Data on the prevalence of AHPFL, symmetry, origin, insertion, innervation, relationship to AIN and MN, morphology and morphometrics was extracted from the included studies. In the event of any discrepancies in the data, authors were contacted by email, if possible, for further information.

### Statistical analysis

Statistical analysis was performed by JR and PP using MetaXL analysis version 2.0 by EpiGear International Pty Ltd (Wilston, Queensland, Australia). Heterogeneity among the studies was assessed using the Higgin’s *I*^2^ test, with values of 25%, 50%, and 75% indicating low, moderate, and high degrees of heterogeneity, respectively. The single-categorical or multi-categorical pooled prevalence was calculated using a statistical model that was in respect to the level of heterogeneity. If the Higgin’s *I*^2^ was <50%, a fixed effects model was used. If the Higgin’s *I*^2^ was >50%, a random effects model was used. When appropriate, subgroup analysis and/or a sensitivity analysis based on the inclusion of only studies with ≥100 limbs was performed, to probe the sources of heterogeneity.

## Results

### Study identification

**Figure 2:**
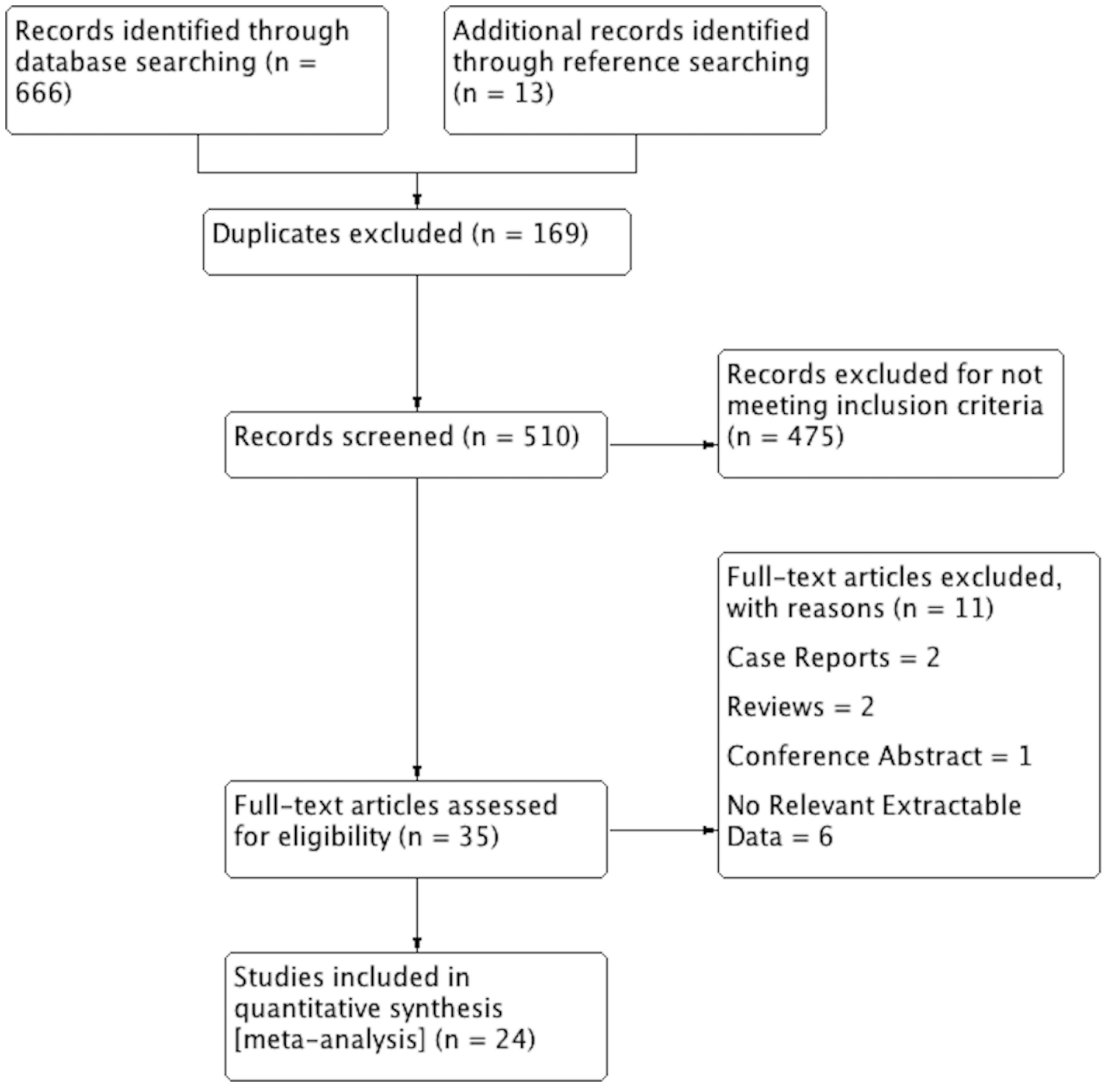
PRISMA flowchart of study identification, evaluation and inclusion in the meta-analysis.

An overview of the study identification process is summarized in Fig. 2. Through extensive database searching, a total of 666 articles were initially identified. Further 13 articles were added through reference searching. Overall, 35 articles were assessed by full text for potential eligibility. Of these, 11 articles were excluded and 24 articles were deemed eligible and included into the meta-analysis. Two articles were excluded for being case reports, two articles were excluded for being reviews, and one conference abstract was also excluded. One study by Alves, Candido & Frazao (2004) was excluded for data only related to the median nerve and the study by Vincelet et al. (2013) was excluded as they presented prevalence data only as a rate per cadaver.

### Characteristics of included studies

The characteristics of included studies is summarized in Table 1, which includes the prevalence of AHFPL in upper limbs as reported by individual studies. Additional study data is provided in Supplemental Information 2. A total of 24 studies (*n* = 2,358 upper limbs) were included in the meta-analysis. The dates of the included studies spanned from 1944 to 2015 (Dykes & Anson, 1944; Caetano et al., 2015; Riveros, Olave & Sousa-Rodrigues, 2015). All studies were performed on adult cadavers, except one study by Kara et al. (2012) who gave individual data on both adults and fetuses. The studies also demonstrated a wide geographical distribution, with the majority of studies hailing from North America, Asia and Europe.

**Table 1:** Characteristics of studies included in the meta-analysis.

Study^*^	Population	*n* (number of upper limbs) =	Number of AHFPL (Prevalence in %)
Mangini (1960)	American	76	56 (73.68%)
Caetano et al. (2015)	Brazilian	80	54 (67.50%)
Hemmady, Subramanya & Mehta (1993)	Indian	54	36 (66.67%)
Oh, Chung & Koh (2000)	Korean	72	48 (66.67%)
Kida (1988)	Japanese	132	82 (62.12%)
Mahakkanukrauh et al. (2004)	Thai	240	149 (62.08%)
El Domiaty, Zoair & Sheta (2008)	Egyptian	42	26 (61.90%)
Shirali et al. (1998)	American	60	33 (55.00%)
Malhotra, Sing & Tewari (1982)	American	240	130 (54.17%)
Dykes & Anson (1944)	American	150	80 (53.33%)
Al-Qattan (1996)	Saudi-Arabian	25	13 (52.00%)
Uyaroglu, Kayalioglu & Erturk (2006)	Turkish	52	27 (51.92%)
Gunnal et al. (2013)	Indian	180	92 (51.11%)
Mori (1964)	Japanese	205	103 (50.24%)
Pai et al. (2008)	Indian	126	58 (46.03%)
Jones et al. (1997)	English	80	36 (45.00%)
Kara et al. (2012) (Adult)	Turkish	52	20 (38.46%)
Dellon & Mackinnon (1987)	Canadian	43	14 (32.56%)
Kara et al. (2012) (Fetus)	Turkish	90	29 (32.22%)
Dolderer et al. (2011)	German	19	5 (26.32%)
Tamang et al. (2013)	Indian	60	15 (25.00%)
Bilecenoglu, Uz & Karalezli (2005)	Turkish	30	6 (20.00%)
Tubbs et al. (2006)	American	20	4 (20.00%)
Riveros, Olave & Sousa-Rodrigues (2015)	Brazilian	30	3 (10.00%)
Sembian et al. (2012)	Indian	200	1 (0.50%)

**Notes.**

* Studies are arranged from highest to lowest prevalence. All studies used only use adult cadavers unless stated otherwise.

### Prevalence

**Figure 3:**
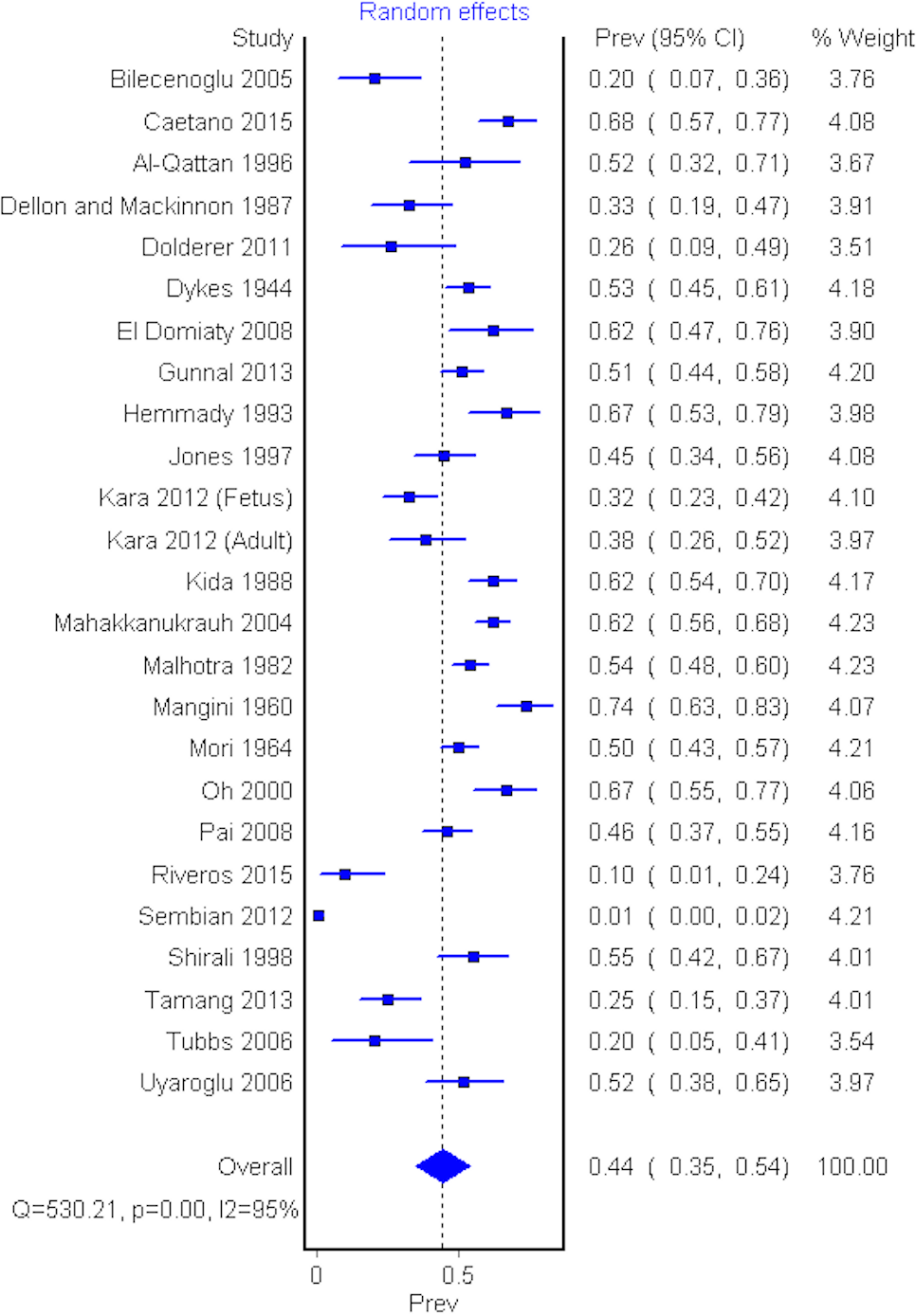
Forrest plot of prevalence of AHFPL.

Twenty-four cadaveric studies (*n* = 2,358 upper limbs) reported the prevalence of AHFPL (Dolderer et al., 2011; El Domiaty, Zoair & Sheta, 2008; Caetano et al., 2015; Kara et al., 2012; Jones et al., 1997; Dykes & Anson, 1944; Gunnal et al., 2013; Mangini, 1960; Hemmady, Subramanya & Mehta, 1993; Dellon & Mackinnon, 1987; Al-Qattan, 1996; Oh, Chung & Koh, 2000; Bilecenoglu, Uz & Karalezli, 2005; Kida, 1988; Mahakkanukrauh et al., 2004; Malhotra, Sing & Tewari, 1982; Mori, 1964; Pai et al., 2008; Riveros, Olave & Sousa-Rodrigues, 2015; Sembian et al., 2012; Shirali et al., 1998; Tamang et al., 2013; Tubbs et al., 2006; Uyaroglu, Kayalioglu & Erturk, 2006). The overall pooled prevalence of an AHFPL in upper limbs was 44.2% (95% CI [0.347–0.540], *I*^2^ = 95.5%) (Fig. 3).

A sensitivity analysis was performed by including only studies with a sample size greater than 100 upper limbs. Eight studies (*n* = 1,473 upper limbs) were included in the sensitivity analysis (Dykes & Anson, 1944; Gunnal et al., 2013; Kida, 1988; Mahakkanukrauh et al., 2004; Malhotra, Sing & Tewari, 1982; Mori, 1964; Pai et al., 2008; Sembian et al., 2012). The pooled AHFPL prevalence in this group was 44.7% (95% CI [0.256–0.646], *I*^2^ = 98.3%), and thus consistent with the result of the overall analysis.

To probe the source of heterogeneity among the studies, the prevalence of AHFPL in different population subgroups was calculated and presented in Table 2. The prevalence of AHFPL was found to be most common in North Americans with a prevalence of 50.3% (95% CI [0.393–0.612]), and least common in Europeans with a prevalence of only 37% (95% CI [0.286–0.458]). However, due to the wide overlapping confidence intervals, there were no statistically significant differences between population subgroups.

The prevalence of AHFPL based on gender and side (left vs. right) are presented in Table 3. Four studies (*n* = 402 upper limbs) described the gender distribution of prevalence of the AHFPL (El Domiaty, Zoair & Sheta, 2008; Caetano et al., 2015; Jones et al., 1997; Sembian et al., 2012). Men more commonly had an AHFPL with a pooled prevalence of 41.1% (95% CI [0–0.894]), while women had a pooled prevalence of only 24.1% (95% CI [0–0.706]).

**Table 2:** Prevalence of an AHFPL in different population subgroups.

Subgroup	# of studies	*n* =	Prevalence (%)	*I*^2^ =
Overall prevalence	24	2,358	44.2% (95% CI [0.347–0.540])	95.5
Asia	10	1,294	44.9% (95% CI [0.270–0.653])	97.9
North America	6	589	50.3% (95% CI [0.393–0.612])	83.6
Europe	5	323	37.0% (95% CI [0.286–0.458])	59.6
Sensitivity analysis >100 limbs	8	1,473	44.7% (95% CI [0.256–0.646])	98.3

**Table 3:** Prevalence of an AHFPL in relation to gender and side.

	Type	*n* =	Prevalence
Gender	Male	256	41.1% (95% CI [0–0.894])
	Female	146	24.1% (95% CI [0–0.706])
Side	Right	472	52.8% (95% CI [0.453–0.603])
	Left	448	45.2% (95% CI [0.357–0.548])

A total of 8 studies (*n* = 920 upper limbs) reported proportions of AHFPL prevalence according to the side of upper limb (*I*^2^ = 59.5%) (El Domiaty, Zoair & Sheta, 2008; Jones et al., 1997; Gunnal et al., 2013; Kida, 1988; Malhotra, Sing & Tewari, 1982; Pai et al., 2008; Shirali et al., 1998; Tamang et al., 2013). The prevalence of an AHFPL in upper limbs on the right side was 52.8% (95% CI [0.453–0.603]), slightly higher, but not significantly different from the prevalence of upper limbs on the left side of 45.2% (95% CI [0.357–0.548]).

### Laterality

Ten studies (*n* = 384 cadavers with an AHFPL) described the rate of unilateral and bilateral occurrence of an AHFPL (*I*^2^ = 77.0%) (El Domiaty, Zoair & Sheta, 2008; Kara et al., 2012; Jones et al., 1997; Gunnal et al., 2013; Oh, Chung & Koh, 2000; Mahakkanukrauh et al., 2004; Malhotra, Sing & Tewari, 1982; Shirali et al., 1998; Tamang et al., 2013; Uyaroglu, Kayalioglu & Erturk, 2006). In 52.1% (95% CI [0.409–0.61]) of cadavers with an AHFPL, the accessory head was observed bilaterally, while in 47.9% (95% CI [0.369–0.591]) of cadavers, an AHFPL was observed only unilaterally.

### Origin & insertion of AHFPL

The prevalence of the various origins and insertions of an AHFPL is presented in Table 4. Seventeen studies (*n* = 858 upper limbs with an AHFPL) reported point of origin of an AHFPL (*I*^2^ = 96.5%) (El Domiaty, Zoair & Sheta, 2008; Caetano et al., 2015; Kara et al., 2012; Gunnal et al., 2013; Mangini, 1960; Hemmady, Subramanya & Mehta, 1993; Dellon & Mackinnon, 1987; Al-Qattan, 1996; Oh, Chung & Koh, 2000; Kida, 1988; Mahakkanukrauh et al., 2004; Malhotra, Sing & Tewari, 1982; Mori, 1964; Sembian et al., 2012; Shirali et al., 1998; Tamang et al., 2013; Uyaroglu, Kayalioglu & Erturk, 2006). The most common origin of an AHFPL was the medial epicondyle of the humerus with a pooled prevalence of 43.6% (95% CI [0.166–0.521]). The second most common origin was from the coronoid process of the ulna with a pooled prevalence of 25.8% (95% CI [0.065–0.369]). In 16.1% (95% CI [0–0.602]) of upper limbs, the AHPFL had a dual origin from the medial epicondyle of the humerus and the coronoid process of the ulna.

**Table 4:** Origin and insertion of the AHFPL.

	Type	Prevalence
Origin	Medial epicondyle of humerus	43.6% (95% CI [0.166–0.521])
	Coronoid process of ulna	25.8% (95% CI [0.065–0.369])
	Dual origin from medial epicondyle and coronoid process	16.1% (95% CI [0–0.602])
	Flexor digitorum superficialis	0.7% (95% CI [0–0.238])
	Muscle fascia	0.2% (95% CI [0–0.199])
Insertion	Flexor pollicis longus	94.6% (95% CI [0.731–1.0])
	Flexor digitorum profundus	5.4% (95% CI [0–0.69])

A total of four studies (*n* = 245 upper limbs with an AHFPL) reported data on the insertion of an AHFPL (*I*^2^ = 95.3%) (El Domiaty, Zoair & Sheta, 2008; Caetano et al., 2015; Al-Qattan, 1996; Mahakkanukrauh et al., 2004). In 94.6% of limbs (95% CI [0.731–1.0]) it was inserted into the flexor pollicis longus muscle, while in 5.4% of limbs (95% CI [0–0.69]) it was inserted into the flexor digitorum profundus muscle.

Furthermore, a total of six studies (*n* = 259 upper limbs) reported data on the location of the insertion of the tendon of AHFPL within the forearm (*I*^2^ = 97.0%) (Caetano et al., 2015; Jones et al., 1997; Gunnal et al., 2013; Pai et al., 2008; Sembian et al., 2012; Tamang et al., 2013). In 57.9% of limbs (95% CI [0.165–0.938]) insertion of the tendon was in the proximal third of the forearm, in 36.4% of limbs (95% CI [0.028–0.780]) it was in the middle third of the forearm, and in 5.7% (95% CI [0–0.315]) of limbs it was in the distal third of the forearm.

### Morphology & morphometrics

Eleven studies (*n* = 560 upper limbs with an AHFPL) described morphology of the AHFPL. The pooled prevalence of the different morphologies is presented in Table 5 (El Domiaty, Zoair & Sheta, 2008; Caetano et al., 2015; Kara et al., 2012; Jones et al., 1997; Gunnal et al., 2013; Oh, Chung & Koh, 2000; Mahakkanukrauh et al., 2004; Pai et al., 2008; Riveros, Olave & Sousa-Rodrigues, 2015; Tamang et al., 2013; Uyaroglu, Kayalioglu & Erturk, 2006). The most common shape was fusiform with a pooled prevalence of 72.0% of limbs (95% CI [0.391–0.879], *I*^2^ = 97.0%). The second most common shape of an AHFPL was slender, with a pooled prevalence of 10.6% of limbs (95% CI [0–0.74]).

**Table 5:** Morphology of the AHFPL.

Morphology	Prevalence
Fusiform	72.0% (95% CI [0.391–0.879])
Slender	10.6% (95% CI [0–0.74])
Triangular	5.0% (95% CI [0–0.184])
Papillary	4.6% (95% CI [0–0.175])
Strap-like	4.1% (95% CI [0–0.165])
Voluminous	2.1% (95% CI [0–0.122])
Voluminous-fusiform	1.6% (95% CI [0–0.108])

A total of 8 studies (*n* = 321 upper limbs with an AHFPL) reported data on morphometric parameters of the AHPFL (El Domiaty, Zoair & Sheta, 2008; Jones et al., 1997; Gunnal et al., 2013; Kara et al., 2012; Oh, Chung & Koh, 2000; Pai et al., 2008; Tamang et al., 2013; Uyaroglu, Kayalioglu & Erturk, 2006). The pooled means of the length of the overall muscle, the tendon, and the muscle body, as well as the pooled mean of the muscle belly width, is presented in Table 6.

**Table 6:** Morphometrics of the AHFPL.

Parameter	Pooled mean length ± SD (mm)
Total length of muscle	78.86 ± 10.94
Total length of tendon	8.53 ± 9.02
Length of muscle belly	72.71 ± 12.43
Width of muscle belly	4.15 ± 1.71

### Innervation

A total of 12 studies (*n* = 481 total upper limbs with an AHFPL) reported data regarding innervation of an AHFPL (*I*^2^ = 74.8%) (Caetano et al., 2015; Kara et al., 2012; Jones et al., 1997; Gunnal et al., 2013; Mangini, 1960; Hemmady, Subramanya & Mehta, 1993; Dellon & Mackinnon, 1987; Al-Qattan, 1996; Pai et al., 2008; Shirali et al., 1998; Tamang et al., 2013; Uyaroglu, Kayalioglu & Erturk, 2006). In most cases, the AIN innervated the AHPFL, with a pooled prevalence 97.3% (95% CI [0.924–0.993]). Rarely, the AHFPL was innervated by the MN, with a prevalence of only 2.7 % (95% CI [0.004–0.067]).

### The relationship of the AHFPL to the AIN

Fifteen studies (*n* = 706 upper limbs with an AHFPL) reported data on the relationship of an AHFPL to the AIN (*I*^2^ = 98.6%) (El Domiaty, Zoair & Sheta, 2008; Caetano et al., 2015; Kara et al., 2012; Jones et al., 1997; Gunnal et al., 2013; Mangini, 1960; Hemmady, Subramanya & Mehta, 1993; Dellon & Mackinnon, 1987; Al-Qattan, 1996; Oh, Chung & Koh, 2000; Mahakkanukrauh et al., 2004; Pai et al., 2008; Shirali et al., 1998; Tamang et al., 2013; Uyaroglu, Kayalioglu & Erturk, 2006). An AHFPL was located most commonly anterior to AIN, with a pooled prevalence of 62.5% (95% CI [0.232–0.870]) of limbs. In other instances, an AHFPL was located posterior to AIN in 17.4% (95% CI [0–0.19]) of limbs, medial to AIN in 8.9% (95% CI [0–0.295]) of limbs, anteromedial to AIN in 7.3% (95% CI [0–0.268]) of limbs, and lateral to AIN in 1.3% (95% CI [0–0.134]) of limbs.

A further two studies (*n* = 105 upper limbs with an AHFPL) described the crossing of the AIN by the AHFPL (*I*^2^ = 96.9%) (Caetano et al., 2015; Oh, Chung & Koh, 2000). In 74.0% (95% CI [0.121–1.0]) of limbs, the AIN was crossed by the belly of the AHFPL, in 14.3% (95% CI [0–0.670]) of limbs the AIN was crossed by the tendon of the muscle, and in 11.7% (95% CI [0–0.624]) of limbs the nerve was observed laterally to the AHFPL.

### The relationship of the AHFPL to the MN

A total of 10 studies (*n* = 347 upper limbs with an AHFPL) reported the relationship of the AHFPL to the MN (*I*^2^ = 0%) (El Domiaty, Zoair & Sheta, 2008; Caetano et al., 2015; Kara et al., 2012; Jones et al., 1997; Mangini, 1960; Hemmady, Subramanya & Mehta, 1993; Al-Qattan, 1996; Pai et al., 2008; Shirali et al., 1998; Tamang et al., 2013). The AHFPL was located mainly posterior to MN, with a pooled prevalence of 98.9% (95% CI [0.964–0.993]) of limbs, and occasionally anterior to MN in 1.9% (95% CI [0.007–0.033]) of limbs.

## Discussion

The reported prevalence of AHFPL in literature varies significantly among studies. As such, the aim of our meta-analysis was to pool together all the available studies with prevalence data on the AHFPL and it’s anatomical characteristics. In our review of the literature, we found prevalence rates ranging from as low as 0.5% (Sembian et al., 2012) to as high as 73.68% (Mangini, 1960). This wide discrepancy is perhaps due to the fusion of the muscle with surrounding superficial flexors, leading to misidentification of the muscle, and a lower reported prevalence rate in some studies (Dykes & Anson, 1944; Bilecenoglu, Uz & Karalezli, 2005).

In our analysis, the pooled prevalence of AHFPL in upper limbs was 44.2%, concordant with the 45% prevalence reported in the study by Dellon & Mackinnon (1987). However, numerous studies reported prevalence rates that were significantly higher, for example 67% in Hemmady, Subramanya & Mehta (1993) and 74% in Mangini (1960). Furthermore, in accordance with several studies in literature (Caetano et al., 2015; Mangini, 1960; Hemmady, Subramanya & Mehta, 1993; Dellon & Mackinnon, 1987; Al-Qattan, 1996; Oh, Chung & Koh, 2000), our results also found that the AHFPL was slightly more common bilaterally in cadavers with a prevalence of 52.1%, than unilaterally, which had a prevalence of 47.9%.

Subgroup analysis based on geographical location, focusing on North America, Europe and Asia revealed that, while not statistically significant, North Americans most frequently had an AHFPL with a prevalence of 50.3%, followed by Asians with a prevalence of 44.9% and was least common in Europeans, who had a reported prevalence of only 37.0%. It is interesting to note that this prevalence discrepancy according to race has also been demonstrated by other authors who reported a prevalence of AHFPL as low as 33.3% (Le Double, 1897) in European Caucasians and as high as 89.3% in the population of people of African descent (Kara et al., 2012). As such, it can be hypothesized that as North America has a significantly higher African American population than Europe, it would also have a higher prevalence of AHFPL in the population, as seen in our meta-analysis. However, as most studies did not report the races of their study population, further studies are required to definitively investigate the link between race and prevalence of AHFPL.

In terms of gender distribution, our results revealed an AHFPL to be more common in men with a prevalence of 41.1%, versus women who had a prevalence of only 24.1%. We postulate that due to the intimate fusion of the AHFPL with surrounding structures and the generally smaller size of female upper limbs as compared to men, the AHFPL could be unidentified and thus underreported in women. Several studies also analyzed the distribution of the AHFPL between right and left upper limbs. According to a study by El Domiaty, Zoair & Sheta (2008) the AHFPL was found 77.7% on the right and only in 50% of cases on the left. This trend has been further echoed by Gunnal et al. (2013) who postulated that right predominance of AHFPL is more common because most of the population is right handed with larger right limbs leading to easier identification during dissections and perhaps underreporting of AHFPL in left limbs. In contrast to these studies, our results, although not statistically significant, showed a slightly higher prevalence rate on the right (52.8%) versus the left side (45.2%) of upper limbs, which was more in line with Kara et al. (2012), who reported the AHFPL to be present in 53% of right upper limbs and in 47% of left upper limbs.

In our study we found the AHFPL originated from the medial epicondyle of the humerus in 43.6% of limbs and from the coronoid process of the ulna in 25.8% of limbs. A dual origin from the medial epicondyle and coronoid process was also seen in 16.1% of limbs. The AHFPL nearly always inserted into the FPL with a 94.6% prevalence, with the tendon of the muscle inserting into the proximal third of the forearm in 57.9% of limbs. This presents cadaveric studies with a more reliable methodology to correctly identify the AHFPL despite its potential fusion to the surrounding flexors. Identifying the insertion first and then tracing it back to identify the muscle will allow for more accurate reporting of the muscle prevalence and reduce the wide discrepancy in prevalence rates seen in literature.

While there is agreement in literature regarding the insertion of the muscle, the origin of AHFPL is still widely debated. This disagreement about the origin seems to be due to the misidentification by authors who might confuse the AHFPL joining with the deep part of the FDS and inserting into the medial epicondyle alone or inserting into the medial epicondyle or coronoid process along with the FDS and forming an arch (Caetano et al., 2015).

Furthermore, it is important to take muscle architecture into consideration, as abnormalities in shape may affect both the function and range of movement of the muscle, with the greatest risk of impingement enforced by fusiform and papillary morphological shapes (Oh, Chung & Koh, 2000; Sembian et al., 2012). Our results showed the AHFPL was fusiform in 72% of limbs, followed by slender in 10.6% of limbs and triangular in 5% of limbs, agreeing with results presented by Mahakkanukrauh et al. (2004) and Pai et al. (2008), but different from those presented by Jones et al. (1997), who reported slender AHFPLs to be most common. Therefore, according to our results, a significant number of AHFPL muscles can be seen as potential causes of nerve entrapment neuropathies based on their morphology alone.

In terms of innervation, the accessory muscle was nearly always innervated by the AIN (97.3%) and very rarely by the MN (2.7%). Interestingly, Jones et al. (1997) reported innervation by MN in 44.4% of cases, and also reported a dual innervation of the AHFPL muscle by AIN and MN. We also found that the muscle was most commonly located in between the AIN posteriorly (62.5%) and MN anteriorly (98.9%). Furthermore, we found that the AIN crosses the muscle belly in 74.0% of cases and is found lateral to the muscle in 11.7% of cases. It can be hypothesized that impingement of the AIN by the muscle causing AINS most commonly occurs when it crosses the muscle belly, especially with concurrent AHFPL hypertrophy, and occurs minimally with lateral crossing of the AIN (Gunnal et al., 2013; Mahakkanukrauh et al., 2004).

Anterior Interosseous Nerve Syndrome is thought to be caused by trauma and structural abnormalities in the forearm (Gunnal et al., 2013). Compression of the AIN leading to paralysis, also called Kiloh-Nevin Syndrome, should be suspected in patients with carpal tunnel syndrome that do not respond to conservative or surgical therapy (El Domiaty, Zoair & Sheta, 2008; Gunnal et al., 2013). Although the role of AHFPL causing nerve entrapment neuropathies is debated over, AIN compression is seen to be more plausible in literature than MN compression (Caetano et al., 2015). However, Shirali et al. (1998) reported that the AHFPL can cause compression of both nerves leading to potential clinical symptoms. Furthermore, a case of incomplete AINS and a case of complete AINS caused by mechanical compression by the AHFPL was reported by Tabib, Aboufarah & Asselineau (2001) and Degreef & De Smet (2004), respectively. To aid in the diagnosis of AINS, manual muscle testing and observation of a pinching movement disability can be performed. However, the most accurate testing method has been shown to be electromyographic testing, which diagnoses 80–90% of cases of AINS (Kara et al., 2012). While the MN is most commonly impinged at its origins with symptoms presenting as weakness in upper extremities, AINS often presents with isolated paralysis of FPL and a characteristic pinch movement impairment of the thumb and index finger (Pai et al., 2008).

Combined with diagnostic tests, the presence of an AHFPL and its topographical and anatomical relationships to surrounding structures, particularly the AIN and MN, are fundamental to understanding the pathomechanism of AINS and providing appropriate therapy (Uyaroglu, Kayalioglu & Erturk, 2006). To avoid iatrogenic injury, the presence of an AHFPL should also be taken into account when performing decompression fasciotomies for compartment syndrome of the forearm and surgeries using an anterior approach to the proximal radius and elbow joints (Hemmady, Subramanya & Mehta, 1993).

Our meta-analysis was limited by the small sample size of some of the included studies, the high heterogeneity among the included studies, and the lack of a method for quality and risk of bias assessment for anatomical meta-analysis. Furthermore, the morphological shapes of the muscle were subject to interpretation of the individual studies and were therefore at a risk of bias. We suspected that the high heterogeneity among the included studies reflects the high variability in the true prevalence of the AHFPL. Lastly, no assessment of publication bias was performed, due to the lack of a reliable method for anatomical prevalence meta-analysis.

## Conclusion

The presence of an AHFPL is common in the population and should be considered more a part of normal anatomy than as an anatomical variant. It is also important to emphasize differences in prevalence rates based on geographical and racial distribution. Identifying the insertion of the AHFPL first and then tracing it back to identify the muscle will allow cadaveric studies to more accurately report the prevalence of the muscle and reduce the wide discrepancy in prevalence rates seen in literature. Due to the significant variations in the origin, shape, and topographical relationship to the AIN and MN of the AHFPL, reliable anatomical knowledge is crucial for the accurate diagnosis and treatment of AINS and other nerve entrapment neuropathies, and to avoid iatrogenic injury during decompression procedures.

## Supplemental Information

10.7717/peerj.1255/supp-1 Supplemental Information 1 PRISMA 2009 checklist

10.7717/peerj.1255/supp-2 Supplemental Information 2 Additional study data

## References

[ref-1] Al-Qattan MM (1996). Gantzer’s muscle: an anatomical study of the accessory head of the flexor pollicis longus muscle. The Journal of Hand Surgery: Journal of the British Society for Surgery of the Hand.

[ref-2] Alves N, Candido PL, Frazao R (2004). Innervation of the pronator teres muscle. International Journal of Morphology.

[ref-3] Bilecenoglu B, Uz A, Karalezli N (2005). Possible anatomic structures causing entrapment neuropathies of the median nerve: an anatomic study. ACTA Orthopaedica Belgica.

[ref-4] Caetano BE, Neto JJS, Vieira LA, Caetano MF, Moraes DV (2015). Gantzer muscle. An Anatomical Study. Acta Ortopédica Brasileira.

[ref-5] Degreef I, De Smet L (2004). Anterior interosseous nerve paralysis due to Gantzer’s muscle. ACTA Orthopaedica Belgica.

[ref-6] Dellon AL, Mackinnon SE (1987). Musculoaponeurotic variations along the course of the median nerve in the proximal forearm. The Journal of Hand Surgery: Journal of the British Society for Surgery of the Hand.

[ref-7] Dolderer JH, Prandl EC, Kehrer A, Beham A, Schaller HE, Briggs C, Kelly JL (2011). Solitary paralysis of the flexor pollicis longus muscle after minimally invasive elbow procedures: anatomical and clinical study of the anterior interosseous nerve. Plastic and Reconstructive Surgery.

[ref-8] Dykes J, Anson BJ (1944). The accessory tendon of the flexor pollicis longus muscle. Anatomical Record.

[ref-9] El Domiaty MA, Zoair MM, Sheta AA (2008). The prevalence of accessory heads of the flexor pollicis longus and the flexor digitorum profundus muscles in Egyptians and their relations to median and anterior interosseous nerves. Folia Morph.

[ref-10] Gunnal SA, Siddiqui AU, Daimi SR, Farooqui MS, Wabale RN (2013). A study on the accessory head of the flexor pollicis longus muscle (Gantzer’s Muscle). Journal of Clinical and Diagnostic Research.

[ref-11] Hemmady MV, Subramanya AV, Mehta IM (1993). Occasional head of flexor pollicis longus muscle: a study of its morphology and clinical significance. Journal of Postgraduate Medicine.

[ref-12] Jones M, Abrahams PH, Sanudo JR, Campillo M (1997). Incidence and morphology of accessory heads of flexor pollicis longus and flexor digitorum profundus (Gantzer’s muscles). Journal of Anatomy.

[ref-13] Kara A, Elvan O, Yildiz S, Ozturk H (2012). Accessory head of flexor pollicis longus muscle in fetuses and adult cadavers and its relation to anterior interosseous nerve. Clinical Anatomy.

[ref-14] Kida M (1988). The morphology of Gantzer’s muscle, with special reference to the morphogenesis of the flexor digitorum superficialis. Kaibogaku Zasshi. Journal of Anatomy.

[ref-15] Le Double AF (1897). Traite des Variations du Systeme Musculaire de l’Homme.

[ref-16] Mahakkanukrauh P, Surin P, Ongkana N, Sethadavit M, Vaidhayakarn P (2004). Prevalence of accessory head of flexor pollicis longus muscle and its relation to anterior interosseous nerve in Thai population. Clinical Anatomy.

[ref-17] Malhotra VK, Sing NP, Tewari SP (1982). The accessory head of the flexor pollicis longus muscle and its nerve supply. Anatomischer Anzeiger.

[ref-18] Mangini U (1960). Flexor pollics longus muscle. Its morphology and clinical significance. Journal of Bone and Joint Surgery. American Volume.

[ref-19] Mori M (1964). Statistics on the musculature of the Japanese. Okajimas Folia Anatomica Japonica.

[ref-20] Oh CS, Chung IH, Koh KS (2000). Anatomical study of the accessory head of the flexor pollicis longus and the anterior interosseous nerve in Asians. Clinical Anatomy.

[ref-21] Pai MM, Nayak SR, Krishnamurthy A, Vadgaonkar R, Prabhu LV, Ranade AV, Janardhan JP, Rai R (2008). The accessory heads of flexor pollicis longus and flexor digitorum profundus: incidence and morphology. Clinical Anatomy.

[ref-22] Riveros A, Olave E, Sousa-Rodrigues C (2015). Anatomical study of the accessory head of the flexor pollicis longus and its relation to the anterior interosseous nerve in Brazilian individuals. International Journal of Morphology.

[ref-23] Sembian U, Srimathi T, Muhil M, Nalina Kumari SD, Subramanian T (2012). A study of the accessory muscles in the flexor compartment of the forearm. Journal of Clinical and Diagnostic Research.

[ref-24] Shirali S, Hanson M, Branovacki G, Gonzalez M (1998). The flexor pollicis longus and its relation to the anterior and posterior interosseous nerves. The Journal of Hand Surgery: Journal of the British Society for Surgery of the Hand.

[ref-25] Tabib W, Aboufarah F, Asselineau A (2001). Compression du nerf interosseux anterieur par le muscle de Gantzer. Chirurgie de la Main.

[ref-26] Tamang BK, Sinha P, Sarda RK, Shailo P, Murtimanju BV (2013). Incidence and morphology of acessory head of flexor pollicis longus muscle. An anatomical study. Journal of Evolution of Medical and Dental Sciences.

[ref-27] Tubbs RS, Custis JW, Salter EG, Wellons JC, Blount JP, Oakes WJ (2006). Quantitation of and superficial surgical landmarks for the anterior interosseous nerve. Journal of Neurosurgery.

[ref-28] Uyaroglu FG, Kayalioglu G, Erturk M (2006). Incidence and morphology of the accessory head of the flexor pollicis longus muscle (Gantzer‘s muscle) in a Turkish population. Neuroscience.

[ref-29] Vincelet Y, Journeau P, Popkov D, Haumont T, Lascombes P (2013). The anatomical basis for anterior interosseous nerve palsy secondary to supracondylar humerus fractures in children. Orthopaedics & Traumatology: Surgery & Research.

[ref-30] Wood J (1868). Variations in human myology. Proceedings of the Royal Society of London. Series B: Biological Sciences.

