# Supplementary material for: The prevalence and anatomical characteristics of the accessory head of the flexor pollicis longus muscle: a meta-analysis
Source: PeerJ. 2015 Oct 1;3:e1255. doi: 10.7717/peerj.1255 (PMC4636409; doi:10.7717/peerj.1255)
Supplement: Supplemental Information 2 [file peerj-03-1255-s002.pdf]

Table 1 – Prevalence of AHFPL

Study	Type of Study	Continent	Country	n= (total # of limbs)	# of AHFPL	Percentage of limbs with AHFPL
**Al-Qattan 1996**	Cadaveric	Asia	Saudi Arabia	25	13	52.00%
**Bilecenoglu 2005**	Cadaveric	Europe	Turkey	30	6	20.00%
**Caetano 2015**	Cadaveric	South America	Brazil	80	54	67.50%
**Dellon and Mackinnon 1987**	Cadaveric	North America	Canada	43	14	32.56%
**Dolderer 2011**	Cadaveric	Europe	Germany	19	5	26.32%
**Dykes 1944**	Cadaveric	North America	USA	150	80	53.33%
**El Domiaty 2008**	Cadaveric	Africa	Egypt	42	26	61.90%
**Gunnal 2013**	Cadaveric	Asia	India	180	92	51.11%
**Hemmady 1993**	Cadaveric	Asia	India	54	36	66.67%
**Jones 1997**	Cadaveric	Europe	England	80	36	45.00%
**Kara 2012 (Adult)**	Cadaveric	Europe	Turkey	52	20	38.46%
**Kara 2012 (Fetus)**	Cadaveric	Europe	Turkey	90	29	32.22%
**Kida 1988**	Cadaveric	Asia	Japan	132	82	62.12%
**Mahakkanukrauh 2004**	Cadaveric	Asia	Thailand	240	149	62.08%
**Malhotra 1982**	Cadaveric	North America	USA	240	130	54.17%
**Mangini 1960**	Cadaveric	North America	USA	76	56	73.68%
**Mori 1964**	Cadaveric	Asia	Japan	205	103	50.24%
**Oh 2000**	Cadaveric	Asia	Korea	72	48	66.67%
**Pai 2008**	Cadaveric	Asia	India	126	58	46.03%
**Riveros 2015**	Cadaveric	South America	Brazil	30	3	10.00%
**Sembian 2012**	Cadaveric	Asia	India	200	1	0.50%
**Shirali 1998**	Cadaveric	North America	USA	60	33	55.00%
**Tamang 2013**	Cadaveric	Asia	India	60	15	25.00%
**Tubbs 2006**	Cadaveric	North America	USA	20	4	20.00%
**Uyaroglu 2006**	Cadaveric	Europe	Turkey	52	27	51.92%

Table 2 – Origin of the AHFPL

Study	Type of Study	Continent	Country	n = (# of limbs w/AHFPL)	Flexor Digitorum Superficialis	Coronoid Process of Ulna	Medial Epicondyle of Humerus	Dual origin from Medial Epicondyle and Coronoid Process	Muscle Fascia from Proximal Forearm
**Caetano 2015**	Cadaveric	South America	Brazil	57	42	8	7	0	0
**Al-Qattan 1996**	Cadaveric	Asia	Saudi Arabia	13	0	0	11	2	0
**Dellon and Mackinnon 1987**	Cadaveric	North America	Canada	14	0	0	14	0	0
**El Domiaty 2008**	Cadaveric	Africa	Egypt	26	10	2	8	6	0
**Gunnal 2013**	Cadaveric	Asia	India	92	2	76	10	4	0
**Hemmady 1993**	Cadaveric	Asia	India	36	2	6	20	8	0
**Kara 2012 (Fetus)**	Cadaveric	Europe	Turkey	29	24	1	4	0	0
**Kara 2012 (Adult)**	Cadaveric	Europe	Turkey	20	9	2	9	0	0
**Kida 1988**	Cadaveric	Asia	Japan	11	0	0	0	0	11
**Mahakkanukrauh 2004**	Cadaveric	Asia	Thailand	149	3	35	111	0	0
**Malhotra 1982**	Cadaveric	North America	USA	130	0	4	54	72	0
**Mangini 1960**	Cadaveric	North America	USA	54	0	12	34	3	5
**Mori 1964**	Cadaveric	Asia	Japan	103	0	70	25	8	0
**Oh 2000**	Cadaveric	Asia	Korea	48	1	42	5	0	0
**Sembian 2012**	Cadaveric	Asia	India	1	1	0	0	0	0
**Shirali 1998**	Cadaveric	North America	USA	33	0	0	0	0	33
**Tamang 2013**	Cadaveric	Asia	India	15	8	0	5	0	2
**Uyaroglu 2006**	Cadaveric	Europe	Turkey	27	0	22	5	0	0

Table 3 – Insertion of the AHFPL

Study	Type of Study	Continent	Country	n = (# of limbs with AHFPL)	Muscle	Tendon
**Caetano 2015**	Cadaveric	South America	Brazil	57	48	9
**Oh 2000**	Cadaveric	Asia	Korea	48	27	5
				105		

Table 4 – Innervation of the AHFPL

Study	Type of Study	Continent	Country	n= (# of limbs w/AHFPL)	Anterior to AIN	Posterior to AIN
**Caetano 2015**	Cadaveric	South America	Brazil	54	18	30
**Al-Qattan 1996**	Cadaveric	Asia	Saudi Arabia	13	0	13
**Dellon and Mackinnon 1987**	Cadaveric	North America	Canada	14	0	14
**El Domiaty 2008**	Cadaveric	Africa	Egypt	26	26	0
**Gunnal 2013**	Cadaveric	Asia	India	92	0	9
**Hemmady 1993**	Cadaveric	Asia	India	36	36	0
**Jones 1997**	Cadaveric	Europe	England	36	36	0
**Kara 2012 (Fetus)**	Cadaveric	Europe	Turkey	29	26	0
**Kara 2012 (Adult)**	Cadaveric	Europe	Turkey	20	15	0
**Mahakkanukrauh 2004**	Cadaveric	Asia	Thailand	149	12	20
**Mangini 1960**	Cadaveric	North America	USA	56	56	0
**Oh 2000**	Cadaveric	Asia	Korea	48	0	32
**Pai 2008**	Cadaveric	Asia	India	58	58	0

Table 5 – Relationship of AHFPL to the Anterior Interosseous Nerve

Study	Type of Study	Continent	Country	n= (# of limbs w/AHFPL)	Anterior to AIN	Posterior to AIN	Lateral to AIN	Anteromedial to AIN	Medial to AIN
**Caetano 2015**	Cadaveric	South America	Brazil	54	18	30	6	0	0
**Al-Qattan 1996**	Cadaveric	Asia	Saudi Arabia	13	0	13	0	0	0
**Dellon and Mackinnon 1987**	Cadaveric	North America	Canada	14	0	14	0	0	0
**El Domiaty 2008**	Cadaveric	Africa	Egypt	26	26	0	0	0	0
**Gunnal 2013**	Cadaveric	Asia	India	92	0	9	0	83	0
**Hemmady 1993**	Cadaveric	Asia	India	36	36	0	0	0	0
**Jones 1997**	Cadaveric	Europe	England	36	36	0	0	0	0
**Kara 2012 (Fetus)**	Cadaveric	Europe	Turkey	29	26	0	0	3	0
**Kara 2012 (Adult)**	Cadaveric	Europe	Turkey	20	15	0	0	4	1
**Mahakkanukrauh 2004**	Cadaveric	Asia	Thailand	149	12	20	0	19	98
**Mangini 1960**	Cadaveric	North America	USA	56	56	0	0	0	0
**Oh 2000**	Cadaveric	Asia	Korea	48	0	32	0	0	12
**Pai 2008**	Cadaveric	Asia	India	58	58	0	0	0	0
**Shirali 1998**	Cadaveric	North America	USA	33	33	0	0	0	0
**Tamang 2013**	Cadaveric	Asia	India	15	0	0	0	0	15
**Uyaroglu 2006**	Cadaveric	Europe	Turkey	27	15	1	0	8	3

Table 6 – Relationship of the AHFPL to the Median Nerve

Study	Type of Study	Continent	Country	n= (# of limbs w/AHFPL)	Anterior to MN	Posterior to MN
**Caetano 2015**	Cadaveric	South America	Brazil	54	2	52
**Al-Qattan 1996**	Cadaveric	Asia	Saudi Arabia	13	0	13
**El Domiaty 2008**	Cadaveric	Africa	Egypt	26	0	26
**Hemmady 1993**	Cadaveric	Asia	India	36	0	36
**Jones 1997**	Cadaveric	Europe	England	36	0	36
**Kara 2012 (Adult)**	Cadaveric	Europe	Turkey	20	0	20
**Mangini 1960**	Cadaveric	North America	USA	56	0	56
**Pai 2008**	Cadaveric	Asia	India	58	1	57
**Shirali 1998**	Cadaveric	North America	USA	33	3	30
**Tamang 2013**	Cadaveric	Asia	India	15	0	15

Table 7 – Morphometrics of the AHFPL

Study	Type of Study	Continent	Country	n= (# of limbs w/AHFPL)	Mean total length of Muscle (mm)	SD	Mean length of Tendon (mm)	SD	Mean length of Muscle Belly (mm)	SD	Mean width of Muscle belly (mm)	SD
**El Domiaty 2008**	Cadaveric	Africa	Egypt	26	74.66	5.97	8.34	1.01	66.32	6.29		
**Gunnal 2013**	Cadaveric	Asia	India	92	80.47	1.01	1.9	0.09			0.78	0.03
**Jones 1997**	Cadaveric	Europe	England	36	80	16	11.7	13	68	17		
**Kara 2012**	Cadaveric	Europe	Turkey	19	82	12.6			74	12	7	2.2
**Oh 2000**	Cadaveric	Asia	Korea	48							7	2.5
**Pai 2008**	Cadaveric	Asia	India	58	80	15						
**Tamang 2013**	Cadaveric	Asia	India	15			20	8.09	93.48	1.76	7.03	1.43
**Uyaroglu 2006**	Cadaveric	Europe	Turkey	27	71.25	11.97	20.7	17.7			6.99	2.38

Table 8 – Morphology of the AHFPL

Study	Type of Study	Continent	Country	n= (# of limbs w/AHFPL)	Fusiform	Voluminous	Slender	Voluminous Fusiform	Triangular	Strap-like	Papillary
**Caetano 2015**	Cadaveric	South America	Brazil	57	57	0	0	0	0	0	0
**El Domiaty 2008**	Cadaveric	Africa	Egypt	26	14	0	8	4	0	0	0
**Gunnal 2013**	Cadaveric	Asia	India	92	77	0	0	0	15	0	0
**Jones 1997**	Cadaveric	Europe	England	36	0	3	20	0	13	0	0
**Kara 2012 (Fetus)**	Cadaveric	Europe	Turkey	29	12	0	8	0	9	0	0
**Kara 2012 (Adult)**	Cadaveric	Europe	Turkey	20	10	0	7	0	0	3	0
**Mahakkanukrauh 2004**	Cadaveric	Asia	Thailand	149	146	0	3	0	0	0	0
**Oh 2000**	Cadaveric	Asia	Korea	48	9	0	0	0	0	9	30
**Pai 2008**	Cadaveric	Asia	India	58	35	7	0	0	0	16	0
**Riveros 2015**	Cadaveric	South America	Brazil	3	3	0	0	0	0	0	0
**Tamang 2013**	Cadaveric	Asia	India	15	13	0	2	0	0	0	0
**Uyaroglu 2006**	Cadaveric	Europe	Turkey	27	19	0	0	0	0	0	8

Table 9 – Unilateral vs Bilateral AHFPL

Study	Type of Study	Continent	Country	n= (# of cadavers with AHFPL)	Bilateral	Unilateral
**El Domiaty 2008**	Cadaveric	Africa	Egypt	18	2	16
**Gunnal 2013**	Cadaveric	Asia	India	59	33	26
**Jones 1997**	Cadaveric	Europe	England	22	14	8
**Kara 2012 (Fetus)**	Cadaveric	Europe	Turkey	19	10	9
**Kara 2012 (Adult)**	Cadaveric	Europe	Turkey	12	8	4
**Mahakkanukrauh 2004**	Cadaveric	Asia	Thailand	85	64	21
**Malhotra 1982**	Cadaveric	North America	USA	88	42	46
**Oh 2000**	Cadaveric	Asia	Korea	30	18	12
**Shirali 1998**	Cadaveric	North America	USA	21	12	9
**Tamang 2013**	Cadaveric	Asia	India	13	2	11
**Uyaroglu 2006**	Cadaveric	Europe	Turkey	17	10	7

Table 10 – Side Distribution of AHFPL

Study	Type of Study	Continent	Country	n = (total # of limbs)	Left	n = (total # of limbs)	Right
**El Domiaty 2008**	Cadaveric	Africa	Egypt	24	12	18	14
**Gunnal 2013**	Cadaveric	Asia	India	90	43	90	49
**Jones 1997**	Cadaveric	Europe	England	40	16	40	20
**Kida 1988**	Cadaveric	Asia	Japan	67	44	65	38
**Malhotra 1982**	Cadaveric	North America	USA	104	52	136	72
**Pai 2008**	Cadaveric	Asia	India	63	26	63	32
**Shirali 1998**	Cadaveric	North America	USA	30	15	30	18
**Tamang 2013**	Cadaveric	Asia	India	30	4	30	7

Table 11 – Gender Distribution of AHFPL

Study	Type of Study	Continent	Country	n = (total # of male limbs)	Male	n = (total # of female limbs)	Female
**Caetano 2015**	Cadaveric	South America	Brazil	76	53	4	1
**El Domiaty 2008**	Cadaveric	Africa	Egypt	36	23	6	3
**Jones 1997**	Cadaveric	Europe	England	44	21	36	15
**Sembian 2012**	Cadaveric	Asia	India	100	1	100	0

Table 12 – Insertion of tendon of AHFPL

Study	Type of Study	Continent	Country	n= (# of limbs w/AHFPL)	Proximal third of Forearm	Middle third of Forearm	Distal third of Forearm
**Caetano 2015**	Cadaveric	South America	Brazil	57	23	20	14
**Jones 1997**	Cadaveric	Europe	England	36	27	8	1
**Pai 2008**	Cadaveric	Asia	India	58	46	10	2
**Gunnal 2013**	Cadaveric	Asia	India	92	92	0	0
**Sembian 2012**	Cadaveric	Asia	India	1	0	1	0
**Tamang 2013**	Cadaveric	Asia	India	15	0	15	0

Table 13 – Crossing of the Anterior Interosseous Nerve in Relation to the AHFPL

Study	Type of Study	Continent	Country	n = (# of limbs with AHFPL)	Muscle	Tendon	Lateral
**Caetano 2015**	Cadaveric	South America	Brazil	57	48	9	0
**Oh 2000**	Cadaveric	Asia	Korea	48	27	5	16
